# At the US Epicenter of the COVID-19 Pandemic, an Orthopedic Residency Program Reorganizes

**DOI:** 10.1007/s11420-020-09765-5

**Published:** 2020-06-30

**Authors:** Kyle W. Morse, Lauren E. Wessel, Ajay Premkumar, Evan W. James, Benedict U. Nwachukwu, Duretti T. Fufa, , on behalf of the Orthopaedic Surgery Residency Program at the Hospital for Special Surgery

**Affiliations:** Department of Orthopedic Surgery, Hospital for Special Surgery, 535 East 70th Street, New York, NY 10021 USA

**Keywords:** residency, education, COVID-19, communication, organization

## Background

Declared a pandemic by the World Health Organization in mid-March 2020, COVID-19, the disease caused by the novel coronavirus (SARS-CoV-2), spread rapidly in New York City, causing such upheaval that many facilities were forced to modify the care they provided in unexpected ways. One example: on March 13, the Hospital for Special Surgery (HSS) was notified that an entire trauma team of nine orthopedic residents had high-risk exposure from a co-worker who tested positive for COVID-19; all nine were quarantined. With this single exposure affecting 20% of the orthopedic residents, our residency program was tasked with restructuring in order to meet the needs of the five institutions we serve, while minimizing the risk to residents of nonessential exposures.

In this article, we share the guiding framework we used in our institutional reorganization, in the hope that it may help other orthopedic programs responding to similar situations in the future. The principles we used included safety to all patients and staff, transparent communication, organized leadership, and teamwork.

## Hospital Operational Reorganization

Like many large orthopedic surgical training programs, the HSS residency provides orthopedic coverage to several area hospitals, each with different needs during the COVID-19 pandemic (Table 1).

**Table 1:** Hospital sites covered by the Hospital for Special Surgery residents and resident coverage before and during the COVID-19 pandemic

Hospital	Location	# of beds*	Resident ortho coverage (traditional)	Resident coverage during COVID-19 pandemic
Hospital for Special Surgery	Manhattan	215	OR, inpatient, clinic	OR, inpatient, triage center/urgent care, ICU
New York Presbyterian/Weill Cornell Medical Center	Manhattan	1156	OR, inpatient, clinic, ED consult	OR, inpatient, ED consult
New York Presbyterian/Queens	Queens	535	OR, inpatient, clinic, ED consult	OR, inpatient, ED consult, ICU
Memorial Sloan Kettering Cancer Center	Manhattan	498	OR, inpatient, clinic, urgent care consult	OR, clinic, inpatient, urgent care consult
James J. Peters Department of Veterans Affairs Medical Center	Bronx	311	OR, inpatient, clinic, ED consult	OR, clinic, inpatient, ED consult

*Note that hospital capacity reflects pre-coronavirus states *ortho* orthopedic, *OR* operating room, *ICU* intensive care unit, *ED* >emergency department

Due to the severity of the pandemic in New York City, which in March 2020 was responsible for well over half the nation’s cases of COVID-19, the unprecedented strain on capacity within area hospitals was tremendous. In turn, the traditional services provided at each of these five hospitals changed dramatically. Across the state, institutions canceled nonessential procedures and patient visits to increase hospital bed capacity in response to COVID-19, with guidance provided from government agencies and medical societies [2, 12]. On March 17, HSS suspended nonessential procedures, and lists of “essential procedures” were developed for each hospital [8]. Essential procedures are time sensitive and cannot be scheduled in an outpatient setting due to possible loss of life or limb [8].

In light of the reduction in orthopedic case volumes, from approximately 120 cases to 15 per day, traditional service-based resident rotations were rapidly shifted in order to achieve each hospital’s service goals. Additionally, there was a decrease in outpatient clinical volume, as HSS implemented telemedicine to reduce disease transmission while providing clinical care. While the residency was not initially involved in its implementation, residents were offered shadowing opportunities across every service to learn how to take a history and perform a physical examination remotely using this technology, which provided valuable educational experience in what will likely become a more widespread practice in many specialties.

Efforts were made to divert all orthopedic care from New York Presbyterian (NYP) affiliates to HSS. One such effort was the establishment of an orthopedic triage center located at HSS and several other satellite locations in the metropolitan area, to decant surrounding emergency department (ED) and urgent care musculoskeletal patients. In addition, given that HSS is unique in managing high orthopedic patient volumes, the hospital began accepting transfers of all orthopedic patients from neighboring hospitals. As the COVID-19 patient volume began increasing at NYP/Weill Cornell Medical Center (NYP/WCMC), HSS began accepting general medicine and general surgery patients.

Ultimately, due to expanding need in early April, HSS repurposed itself as a COVID-19 facility by increasing its intensive care unit (ICU) capacity in two phases and by converting operating rooms and post-anesthesia care units (PACUs) into 30 critical care beds with negative pressure environments. To accomplish the conversion, the engineering and facilities department constructed walls in the PACU and rerouted the heating, ventilation, and air conditioning system throughout the floor, which took over a week to complete. HSS orthopedic residents not only worked in the orthopedic trauma center but also began assisting anesthesia colleagues in the ICU, as has been seen in other programs [14].

## Shifts in Communication Culture

Communication failure is common during times of unexpected change [4]. Communication is a vital component of an organization’s structure; beyond an exchange of information, it strengthens teams, builds trust, and develops understanding [6, 12]. The challenge of effective communication and the role of the orthopedic surgeon has been studied previously for error prevention [10, 20]. In situations such as a pandemic, similar principles can be established to improve the flow of communication.

Early on in the pandemic and during the first phase of the residency’s reorganization, we realized that we would need an enhanced system for communication to meet the challenges of providing care at outside hospitals, address the concerns of each residency class, and reduce misinformation [4]. Prior to the pandemic, information was disseminated within the residency in large groups; this was no longer possible given isolation restrictions and the rapid changes wrought by the pandemic.

**Fig. 1:**
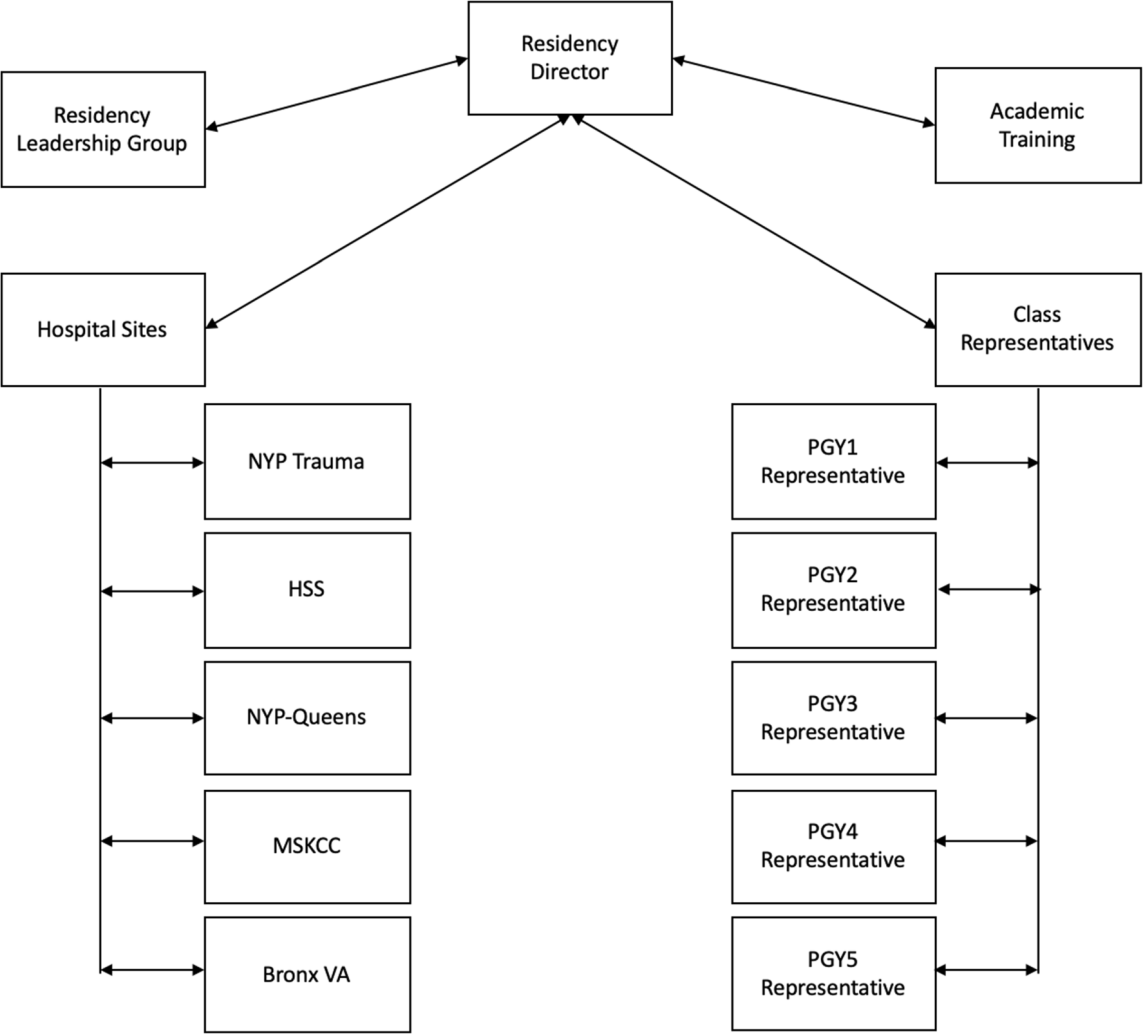
Organization of the COVID-19 Resident Representative Group. *NYP Trauma* New York Presbyterian/Weill Cornell Medical Center, *PGY* post-graduate year, *HSS* Hospital for Special Surgery, *NYP-Queens* New York Presbyterian/Queens, *MSKCC* Memorial Sloan Kettering Cancer Center, *Bronx VA* James J. Peters Department of Veterans Affairs Medical Center

We established a COVID-19 Resident Representative Group consisting of a class-designated representative from each post-graduate year, the resident service chief at each hospital site, and members from both Academic Training and the faculty Residency Leadership Group (RLG) (Fig. 1). The group is chaired by the residency director. The group formalized a horizontal and vertical communication structure, which served to maintain order and efficiency on critical update phone conferences. The horizontal portion of the communication structure occurred within each residency class through both the respective representative and the clinical site. Through the class representative, residents were able to express concerns specific to their post-graduate year. Additionally, the format allowed clinical site chiefs to represent specific concerns regarding their resident team and hospital site. The information passed vertically to allow decision makers to gain perspective in real time. The link to each residency class also allowed the RLG to communicate directly to residents and provide a reliable flow of information.

A group teleconference was held with these personnel every other night. Success of the meetings depended on several factors. First, an agenda was strictly adhered to with dedicated speakers for each topic, and the format of each teleconference was the same from call to call. This agenda included (1) relevant hospital-wide updates since the last call, (2) updates from each clinical site resident chief, and (3) updates from each resident class representative. Specific subjects covered included health status of personnel and relevant quarantines, number of consults performed, number of patients seen, ED/COVID exposures, available personal protective equipment (PPE), and other issues needing to be addressed. A personnel-tracking system was created with a shared document reflecting this information on a running basis. The service chiefs, class representatives, and administrative support staff updated the document daily to enable rapid workforce shifts. Action items were distributed and tasks assigned at the meeting’s conclusion. Minutes were sent following the meeting.

Several other hospital-wide lines of communication were also established. In addition to the residency-led group, the HSS COVID-19 Leadership Group held a semiweekly livestream video conference with all of the residents, fellows, and medical staff to deliver new information and answer questions. A cellphone- and web-based application was also created and consistently updated to provide critical information about hospital policy on COVID-19. Last, emergent communications were distributed by residency leaders for matters pertaining to the residency, such as changes in PPE status, resident health status, or tasks and roles. The frequency of communication and variety of modalities have led to constant access to information. We provided these resources in an effort to decrease anxiety and confusion created by the unknown [13].

## Resident Team Structure and Aims

All orthopedic residents and class representatives worked together to create an equitable system for covering each of the five hospitals. Each hospital’s rapidly changing needs and clinical care burdens were assessed through the group teleconferences. Through class representatives, the orthopedic residency classes worked to determine personnel needs as the number of residents and team structure were modified for each hospital, with the aim of promoting resident and patient safety while meeting patient care needs.

This approach involved rotating resident teams among clinical sites. We made the teams as lean as possible; each role was filled by a resident with appropriate training. With the aim of providing orthopedic trauma care at each clinical site and critical care at HSS and NYP/Queens (Table 1), while minimizing undue SARS-CoV-2 exposure for residents, we adopted an approach of shared responsibility, in which senior residents could also function in more traditionally junior resident “on-call” roles. This sharing of exposure risk and workload among residents was founded on the ethical principles of justice, equity, and solidarity. It also eased self-quarantine after high-risk exposure and the self-monitoring of symptoms before return to the hospital. While the intervals between high-risk exposures were variable, depending on whether residents’ exposure or development of COVID-19 symptoms required their removal from the workforce, we created a scheduling template that allowed for the longest possible periods between clinical responsibilities (with the greatest self-quarantine periods being reserved for roles carrying the highest exposure risk). This approach, initially guided by evidence showing that 97.5% of persons who show symptoms after COVID-19 exposure will do so within 11.5 days, was modified as workforce health allowed [11]. Similarly, other residency programs structured their care teams to minimize exposure, using larger teams to account for personnel who become symptomatic and to maintain continuous care as self-quarantine of residents was needed [9, 23]. Social distancing among the residents was also reinforced, especially as all HSS residents live in the same building. Residents who became symptomatic were self-quarantined and referred to Occupational Health Services. Their health status and symptoms were tracked to determine return to work and schedule shifts as needed.

PPE was used for all patient encounters, as indicated by hospital and federal guidelines. Each resident team reported available PPE during the RRG teleconferences to ensure safety and the conservation of supplies. PPE accountability was an early focus due to the potential for critical shortages and for accelerated use during disease burden peaks.

**Fig. 2:**
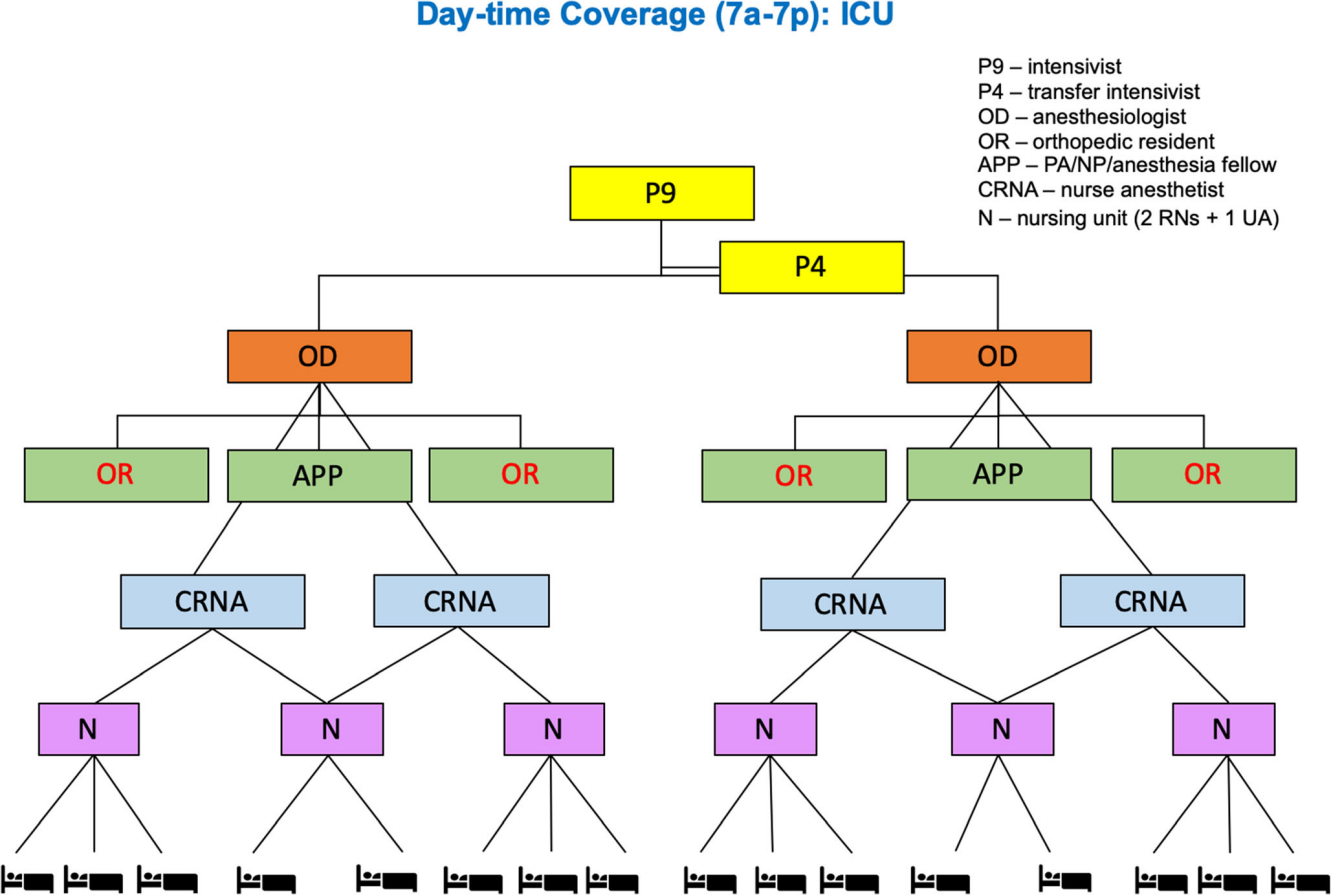
Daytime organization for the Hospital for Special Surgery intensive care unit

**Fig. 3:**
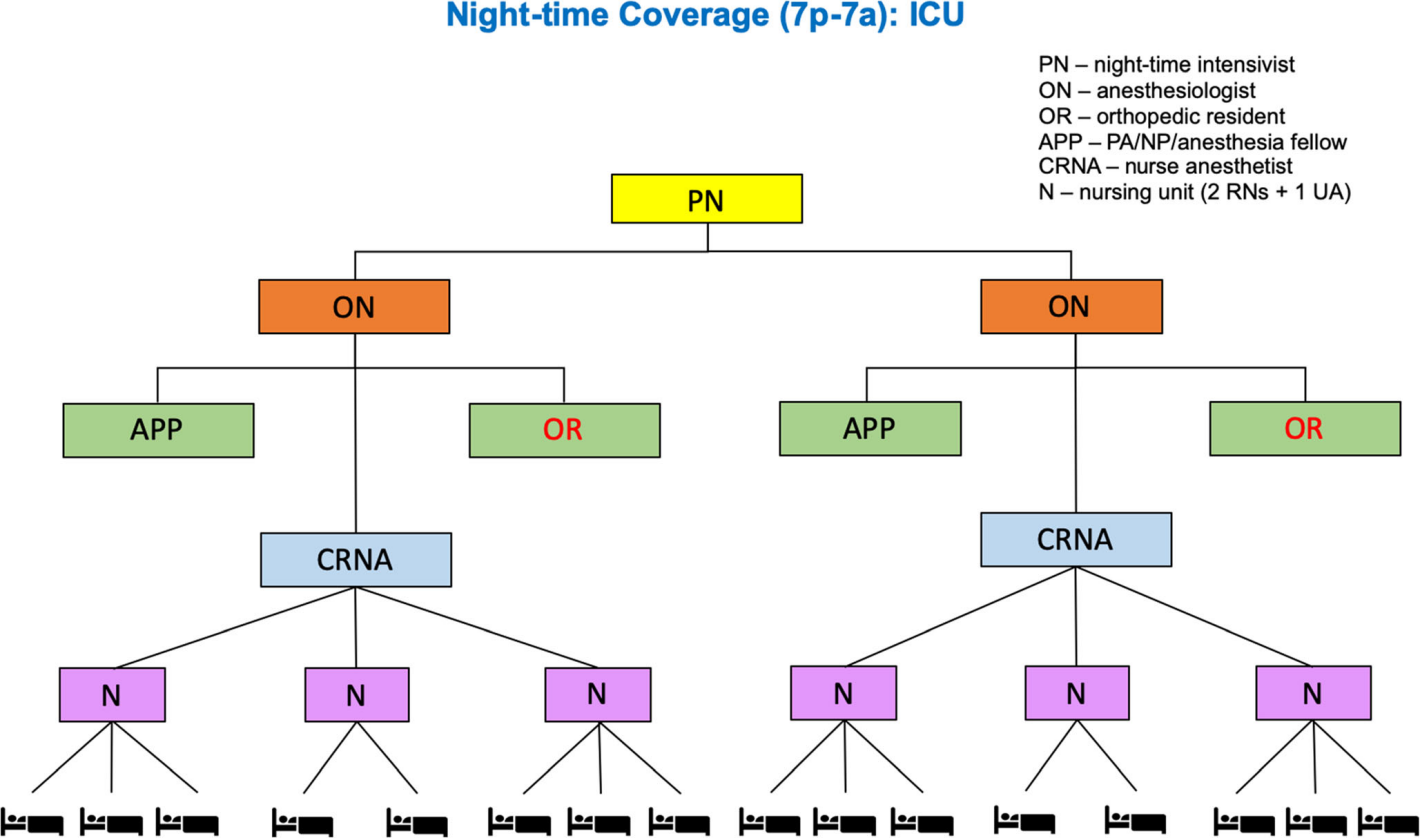
Nighttime organization for the Hospital for Special Surgery intensive care unit

Residents moved between clinical care shifts and sites as needed, facilitated by the transparent communication of clinical needs and workforce health. During the peak case volume periods (the “surge”), when COVID-19 cases surpassed the city’s hospital system reserve capacity, orthopedic residents could volunteer to work outside of established clinical care teams for deployment to the sites of greatest need, including the ED, COVID-19 medical units, and ICUs at both NYP and HSS. As the NYP/WCMC and NYP/Queens hospitals progressed toward 100% COVID-19 patient populations, personnel shifted in order to help meet the desperate need for physicians in these centers but also to provide emergent orthopedic services to those who required them. Prior to the reorganization to meet this need, the RLG and residents reviewed the needs for orthopedic and critical care at each institution. Rotations were assigned to distribute exposure risk, with residents prioritizing their assignments according to their choice; many volunteered for ICU work (Figs. 2 and 3). In addition, the residency program’s teaching faculty transitioned to a focus on the care of COVID-19 patients by maintaining the orthopedic trauma center, working with anesthesiologists and internists, and serving in their traditional teaching capacities in an evolving educational landscape.

## Remote and Self-directed Orthopedic Education

Significant changes to orthopedic graduate medical education (GME) have been required in the USA [15] and worldwide [5] in response to the COVID-19 pandemic. The Accreditation Council for Graduate Medical Education (ACGME) recognized the impact on residency and fellowship accreditation and education of disruptions in surgical training, including a lower case log volume [12]. We implemented changes to GME guided by a conceptual framework put forth by the ACGME, which defined the following three stages of medical education during the COVID-19 pandemic [1].

- Stage 1: Business as usual. Continuation of own specialty patient care and educational activities while preparing for possible increased clinical demands.
- Stage 2: Increased clinical demands. The shift of some residents to pandemic-related patient care activities and suspension of some educational activities.
- Stage 3: Pandemic emergency status. Deployment of most or all residents to pandemic-related patient care activities and suspension of the majority of educational activities.

Educational programming changed rapidly in response to infection control mandates, such as social distancing, and the increasing demands of pandemic-related patient care.

A resident-led task force adapted educational programming, proposing changes to didactic lectures, surgical simulation, journal club, additional funding for access to boards review courses sponsored by the American Academy of Orthopaedic Surgeons and other subspecialty societies, and pandemic-related education, reflecting the severity of disease burden at any given time **(**Table 2). The resident task force also streamlined communication among residents, medical staff, and education leadership. A representative from the education task force participated in all COVID-19 Resident Representative Group calls to ensure that education remained well integrated and reflected changing clinical demands.

**Table 2:** Residency education curriculum by stage, according to Accreditation Council for Graduate Medical Education guidelines

Educational domain	Stage 1: “Business as usual”	Stage 2: Increased clinical demands	Stage 3: Pandemic emergency status
Lectures	In-person general orthopedic and subspecialty lectures	Remote virtual general orthopedic and subspecialty lectures	Optional remote virtual orthopedic and critical care lectures
Bioskills	In-person cadaveric, surgical simulator, and virtual reality training	In-person and remote surgical simulator and virtual reality training	Remote simulation and virtual reality training
Journal Club	In-person large group, residency-wide journal club	Remote virtual small group journal club	Remote virtual small group journal club
Courses	Travel to selected boards review and society courses	Online boards review and society courses	Online boards review and society courses
Pandemic Medicine and Critical Care	None	None	Multidisciplinary resources provided for self-directed learning

Technology was the cornerstone of the newly created curriculum. In-person didactic lectures were suspended indefinitely and replaced with virtual lectures [9]. A virtual weekly calendar was distributed to allow selection of lectures from a menu of offerings across multiple subspecialties. The lectures were recorded and stored on an internal website for residents to access on demand after meeting their patient care responsibilities. In the spirit of PPE stewardship, all cadaveric bioskills education was suspended. Simulation-based learning using, for example, arthroscopy simulators and virtual reality systems was continued on an individual basis, and appropriate cleaning supplies were provided after use [3]. Journal club transitioned from a large group, in-person format to a virtual small group format. Funds usually used to support residents’ travel to society- and industry-sponsored instructional courses and boards review courses were appropriated for virtual courses, such as boards review courses or society-sponsored continuing medical education courses. This allowed for a structured, self-directed study that could be completed as clinical duties permitted.

When the residency program declared Stage 3 status in early April 2020, the educational curriculum changed once again in the face of the increasing demands of pandemic-related patient care. The enhanced virtual didactic lecture schedule continued, but access was limited to those not on active clinical duty. Journal club continued remotely. Simulation-based learning was limited to participation in virtual programs at home on a personal computer or handheld device. Residents continued self-directed study using online boards review and virtual courses, as clinical demands permitted. Most notably, numerous resources regarding COVID-19 infections and care of critically ill patients were compiled and distributed to residents who were deployed to the front lines of pandemic-related patient care. In addition to self-directed learning, grand rounds featuring pulmonologists and critical care medical personnel were held to teach COVID-19 care. The use of technology has been widespread during the pandemic at our institution, and we have allowed for a learning period for its expected continued use [16].

## Well-being and Culture

Managing morale and wellness was paramount during this time period. Recognizing this, HSS made wellness offerings daily, and the American Medical Association released a series of guiding principles with regard to resident wellness, which the residency program strictly adhered to [7]. Additional resources made available to residents included free access to a clinical psychologist and to several virtual mental health programs: NYC Well, Crisis Text Line, Talkspace, and Amwell. In advance of the expected peak of COVID-19 patient volume, the hospital also recruited a chief of staff, crisis management, to add to the emotional and psychological support structures and safety nets for frontline workers. A resident peer support system with formalized training was also established. While the emotional burden associated with sustained exposure to high-risk and high-mortality patient care may not be fully appreciated until some time in the future, residents made use of many of these resources and fared well. High morale was maintained during this time period.

Residents were actively involved in the planning process for patient care and residency team roles through the COVID-19 resident representative group (RRG). Residents freely discussed PPE shortages of and issues with personal protection, all of which were corrected expediently. Instruction was given for the proper donning, doffing, and disposal of PPE. Residents underwent training for preparation in their non orthopedic clinical care roles in the ICU. The RLG, an attending physician, and the chief of crisis management personally called residents who had had exposures. There were weekly videoconference meetings with the resident peer support system, which were well received by the residents. As planning and support for the expected impact on resident well-being remained a top priority of hospital leadership, peer counseling training (4 h) was obtained by three members of the RLG and three residents elected by their peers. Ultimately, because of its culture of cohesion and service, the residency has remained unified through this challenging period.

## Summary

The arrival of the COVID-19 pandemic in the USA, with New York City as its epicenter, has brought unprecedented stresses to the healthcare system. As important members of the frontline workforce, residents disproportionally face these stresses. Given the unique role of residents as both clinicians and trainees, residency and hospital leadership must ensure their health, safety, and well-being amid crisis. In this article, we have outlined the principles we used to guide our residency’s experience at the center of the pandemic. The impressive resident response outlined here was facilitated by the following:

- An organized and committed faculty and resident leadership structure ensuring representation of residents across all levels of training.
- Transparent and frequent communication about the rapidly changing clinical landscape.
- Flexibility and responsiveness regarding both clinical and personal concerns.
- Preservation of resident autonomy in decision making and leadership.

Despite a reduction in orthopedic surgical volume and changes to our educational curriculum, HSS orthopedic residents gained valuable skills in leadership and crisis management, while continuing their education through remote and self-directed learning. That HSS transitioned from a specialty center of musculoskeletal health to a hospital with expanded critical care and general medicine capacity presented an extraordinary challenge. The residents adapted to these challenges and assisted in delivering critical care to patients with COVID-19.

## Electronic supplementary material

ESM 1 (PDF 1225 kb)

ESM 2 (PDF 1224 kb)

ESM 3 (PDF 1224 kb)

ESM 4 (PDF 1224 kb)

ESM 5 (PDF 1224 kb)

ESM 6 (PDF 1224 kb)

## References

[1] Accreditation Council for Graduate Medical Education (ACGME). ACGME response to pandemic crisis. Available from https://www.acgme.org/covid-19. Accessed April 2, 2020.

[2] Amid ongoing COVID-19 pandemic. Governor Cuomo accepts recommendation of Army Corps of Engineers for four temporary hospital sites in New York. [Press release]. March 22, 2020. https://www.governor.ny.gov/news/amid-ongoing-covid-19-pandemic-governor-cuomo-accepts-recommendation-army-corps-engineers-four. Accessed 26 April 2020.

[3] Blumstein G, Zukotynski B, Cevallos N, et al. Randomized trial of a virtual reality tool to teach surgical technique for tibial shaft fracture intramedullary nailing. *J Surg Educ* 2020. S1931-720:30002 10.1016/j.jsurg.2020.01.002. 10.1016/j.jsurg.2020.01.002 PMC7351249 32035854

[4] Brevard SB, Weintraub SL, Aiken JB (2008). Analysis of disaster response plans and the aftermath of Hurricane Katrina: lessons learned from a level I trauma center. J Trauma.

[5] Chang Liang Z, Wang W, Murphy D, Po Hui JH. Novel coronavirus and orthopaedic surgery: early experiences from Singapore. *J Bone Joint Surg Am* 2020. 10.2106/JBJS.20.00236. 10.2106/JBJS.20.00236 PMC7141583 32379113

[6] Ford C (2015). Army leadership and the communication paradox. Mil Rev.

[7] Guiding principles to protect resident and fellow physicians responding to COVID-19. [Press release]. April 13, 2020. https://www.ama-assn.org/delivering-care/public-health/guiding-principles-protect-resident-fellow-physicians-responding. Accessed 26 April 2020.

[8] Hospital for Special Surgery (HSS). Essential Care. 2020. Available from https://www.hss.edu/essential-surgeries-and-care.asp. Accessed April 26, 2020.

[9] Kogan M, Klein SE, Hannon CP, Nolte MT. Orthopaedic education during the COVID-19 pandemic. *J Am Acad Orthop Surg* 2020. 10.5435/JAAOS-D-20-00292. 10.5435/JAAOS-D-20-00292 PMC7195844 32282439

[10] Kuo CC, Robb WJ (2013). Critical roles of orthopaedic surgeon leadership in healthcare systems to improve orthopaedic surgical patient safety. Clin Orthop.

[11] Lauer SA, Grantz KH, Bi Q, et al. The incubation period of coronavirus disease 2019 (COVID-19) from publicly reported confirmed cases: estimation and application. *Ann Intern Med* 2020. 10.7326/M20-0504. 10.7326/M20-0504 PMC7081172 32150748

[12] Potts JR. Residency and Fellowship program accreditation: effects of the novel coronavirus (COVID-19) pandemic. *J Am Coll Surg* 2020. 10.1016/j.jamcollsurg.2020.03.026. 10.1016/j.jamcollsurg.2020.03.026 PMC7194857 32251848

[13] Reynolds B. Crisis and emergency risk communication: by leaders for leaders. Available from https://emergency.cdc.gov/cerc/resources/pdf/leaders.pdf. Accessed April 3, 2020.

[14] Sarpong NO, Forrester LA, Levine WN. What’s important: redeployment of the orthopaedic surgeon during the COVID-19 pandemic: perspectives from the trenches. *J Bone Joint Surg Am* 2020. 10.2106/JBJS.20.00574. 10.2106/JBJS.20.00574 PMC7224625 32287087

[15] Schwartz A, Wilson J, Boden S, Moore T, Bradbury T, Fletcher N. Managing resident workforce and education during the COVID-19 pandemic. *JBJS Open Access*. 2020;[in press]. 10.2106/JBJS.OA.20.00045. 10.2106/JBJS.OA.20.00045 PMC7408274 33117955

[16] Stambough J, Curtin B, Gililland J, et al. The past, present, and future of orthopedic education: lessons learned from the COVID-19 pandemic. *J Arthroplast* 2020. 10.1016/j.arth.2020.04.032. 10.1016/j.arth.2020.04.032 PMC7166110 32345564

